# Mouse models for enteral and parenteral nutrition after surgery

**DOI:** 10.3389/fvets.2025.1626574

**Published:** 2025-09-19

**Authors:** Can Kong, Dingsheng Kong, Junjie Hu, Lianggong Liao, Zhiguo Xiong, Tao Fu

**Affiliations:** ^1^Department of Gastrointestinal Surgery, Hubei Cancer Hospital, Tongji Medical College, Huazhong University of Science and Technology, Wuhan, Hubei, China; ^2^Department of General Surgery, Xi'an Eighth's Hospital, Xi'an, Shaanxi, China; ^3^Department of General Surgery, Qingdao Municipal Hospital, Qingdao, Shandong, China

**Keywords:** total parenteral nutrition, enteral nutrition, mouse model, intestinal barrier, ZO-1

## Abstract

**Background:**

This study aims to establish a mouse model with different nutritional support modalities [enteral nutrition (EN) and parenteral nutrition] after abdominal surgery, providing a stable and effective animal model for further study on the intestinal barrier damage caused by total parenteral nutrition.

**Methods:**

Twenty mice were randomly assigned to either an enteral nutrition (EN) group (*n* = 10) or a total parenteral nutrition (TPN) group (*n* = 10). After abdominal surgery via appendectomy, the two groups of mice received different modes of nutritional support (TPN or EN). A properly sized central venous catheter was placed in the right internal jugular or an enteral nutrition catheter was inserted into the duodenum. Parenteral or enteral nutrition was infused through these catheters. The survival rate, intestinal villus height, epithelial cell proliferation, changes in goblet cells and Paneth cells, as well as the expression levels of the tight junction (TJ) proteins ZO-1 and occludin, were observed.

**Results:**

No difference was found in survival rate (*P* < 0.01) between the two groups. However, significant differences were observed in the height of small intestinal villi, epithelial proliferation rate, number of goblet cells [Periodic Acid-Schiff (PAS+)], Paneth cell function (Lysozyme+), and expression of ZO-1 and occludin proteins. All of these markers were significantly decreased in the TPN group compared to the EN group (*P* < 0.05).

**Conclusion:**

The mouse model is suitable and effective for investigating the pathogenesis of intestinal barrier dysfunction, as it provides different types of nutritional support after abdominal surgery.

## Introduction

Parenteral nutrition (PN) refers to the intravenous administration of nutrients containing the main components of food to meet the nutritional needs of patients who cannot tolerate enteral feeding (1). PN is widely applied as a therapeutic approach in various clinical scenarios, from short-term support in patients with gastrointestinal dysfunction to long-term use in patients with short bowel syndrome (2). Since the demonstration in Beagles in 1966 that total parenteral nutrition (TPN) can provide sole nutritional support for growth and metabolism, research and applications on TPN have expanded rapidly, saving countless critically ill patients (3, 4). Despite the lifesaving benefits of TPN, patients with TPN face a significantly increased risk of infectious complications due to intestinal nutrient deprivation (5–7). Although the precise etiology underlying this increased infection rate remains unclear, many infections are thought to originate from the intestinal flora, suggesting loss of intestinal epithelial barrier function (EBF) associated with TPN may be the cause of these enterally derived infections (8–10). Such gut-derived infections are different from catheter-derived infections caused by TPN. The former mainly results from the loss of intestinal mucosal integrity, while the latter is usually due to bacterial contamination at the puncture site.

Previous studies have reported intestinal villus atrophy, one of the key pathological changes in the intestinal epithelium, which often occurs in TPN patients, leading to impairment of mucosal epithelial barrier function, decreased size of the crypt/villi complex, and reduced intestinal length (11, 12). In contrast, EN significantly reduces the incidence of infectious complications. This protective effect is hypothesized to result from the preservation of intestinal mucosal barrier function, which limits bacterial translocation. Nevertheless, the exact molecular mechanisms underlying this pathological change remain to be fully elucidated (2). Therefore, it is essential to develop an effective animal model for future studies.

Among mammalian experimental animals, mice are widely used in basic research due to their small size, ease of maintenance and handling, short reproductive cycle, and in-depth research background. Thus, this study aims to construct a stable and effective mouse model of TPN and EN for relevant studies.

## Material and methods

The animal study was approved by the Experimental Animal Ethics Committee of Renmin Hospital of Wuhan University (IACUC Issue No. WDRM-20020401A).

### Animals

ICR male specific-pathogen-free mice, aged 8 weeks and weighing 22–25 g, were provided by the Animal Experimental Center of Renmin Hospital, Wuhan University. The mice were housed under controlled humidity, temperature, and lighting conditions. They were allowed to acclimate for 5 days before experimentation and then randomly allocated to receive either EN via enteral nutritional suspension (TPF; *N* = 10) or TPN with intralipid (*N* = 10).

### Surgery

The mice were anesthetized with a mixture of isoflurane and oxygen (1%−3% concentration used, 5% concentration during the induction period). Under aseptic conditions, a laparotomy was performed through a lower midline incision measuring 3 cm. The abdominal wall was retracted with tissue holding forceps, and the right lower abdomen was explored; the cecum was lifted to the incision. The cecal tract was clamped with two separate hemostatic forceps, and the tract was cut short between the hemostatic forceps and ligated separately. Approximately 1 cm from the end of the cecum, the cecum was compressed with hemostatic forceps. The cecum was transected 0.2 cm from the distal end after ligation with a silk thread along the pressed area, and the severed end was disinfected.

### TPN model

The protocol was based on previous studies, with some modifications (13–15). Animals were immobilized as described under the “Surgery” Section. In brief, the mice were anesthetized with a mixture of isoflurane and oxygen (1%−3% concentration was used, with 5% concentration during induction), and then immobilized by securing their limbs on the operating table.

**Figure 1:**
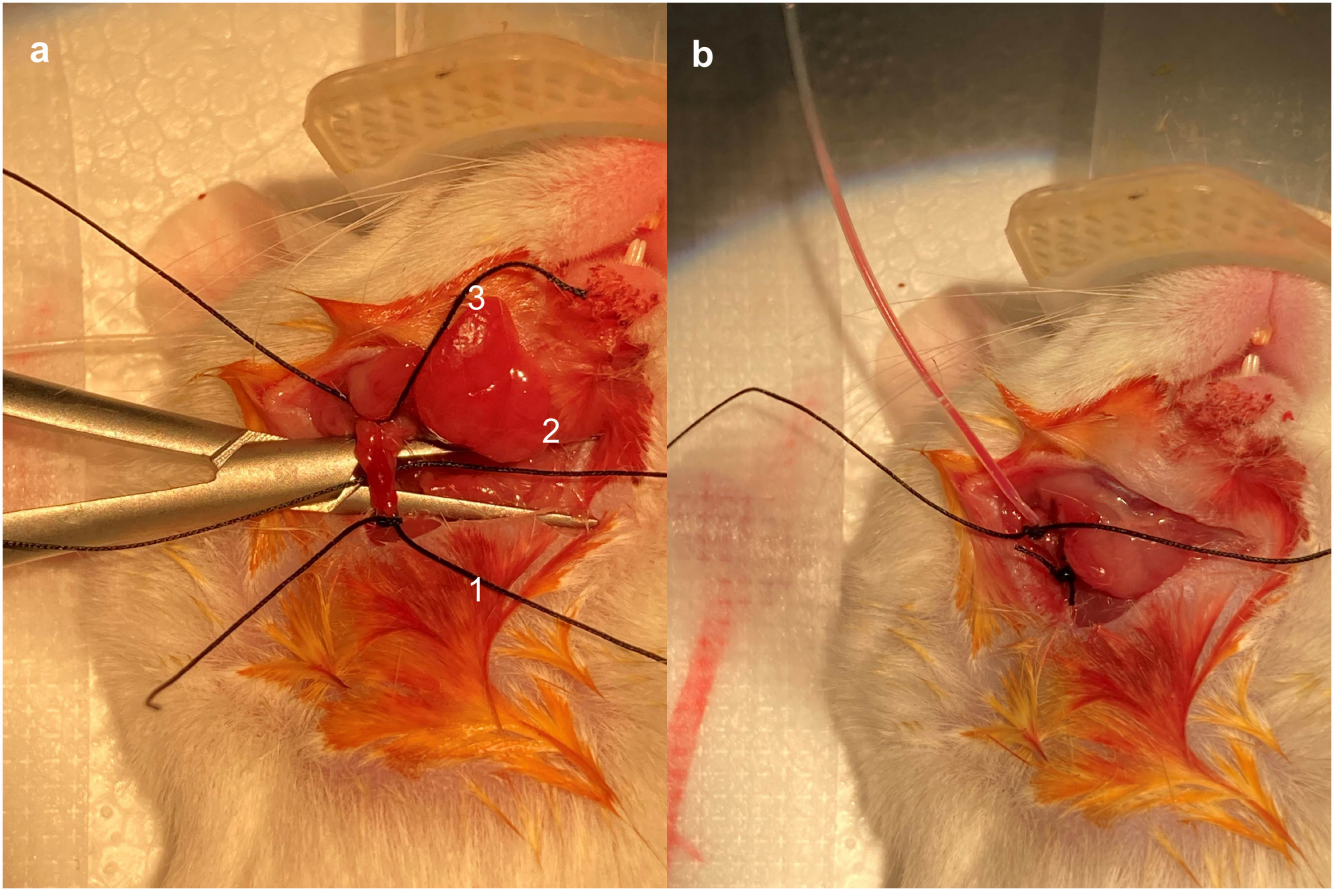
Placement and fixation of central venous catheters. The right jugular vein was identified and skeletally isolated. Three 4-0 silk wires were wrapped under the jugular vein and marked as No. 1, No. 2, and No. 3, respectively, starting from the proximal end **(a)**. Pump back the syringe to make sure blood was returning smoothly **(b)**.

The right cervical region was disinfected, and a vertical median incision was made approximately 1 cm above the clavicle. The incision measured approximately 1 cm in length. The right submandibular gland was identified, and the lateral tissue of the submandibular gland was bluntly separated. The right jugular vein was then identified and carefully isolated. Three 4-0 silk sutures were placed under the jugular vein and marked as No. 1, No. 2, and No. 3, respectively, starting from the proximal end (Figure 1a). The No. 3 suture was tied securely. The vein was gently lifted using vascular forceps to apply some tension. A loose knot was then made in the No. 1 suture without tightening it. While lifting the external jugular vein with flat forceps in one hand, a small incision was made, and the catheter was grasped with small forceps. The catheter (Silastic 60-011-01) was inserted with a small clamp (approximately 0.2 cm from the catheter tip) while the other hand was used to reduce tension and locate the incision based on the bleeding area. The catheter was carefully inserted. Once the catheter tip had passed the No. 1 suture, the No. 1 suture was tied securely. A syringe was pumped back to ensure that blood returned smoothly (Figure 1b). If blood flow was not smooth, the No. 1 suture could be loosened slightly, and the catheter position adjusted. After the blood returns smoothly, tie the No. 2 suture securely again at the proximal end of the incision, where additional knockout knots can be added to enhance fixation. The syringe was aspirated again to ensure a smooth blood return, and the excess suture material was removed.

Fixed catheter: a section of fine suture was threaded into the distal end of the catheter, and then the other end of the fine suture was threaded subcutaneously from the neck of the mouse to the back and then to the tail. The catheter was pulled through the tail to ensure there were no wrinkles. The mouse's tail was fixed onto the foam pad or the iron sheet. The fine suture was withdrawn, and the syringe was connected to the catheter in order to draw blood smoothly.

#### Incision handling

The incision was sutured in two layers—the tendon of the platysma myroides layer and the skin—using 4-0 silk thread, and the area was then cleaned with 75% ethanol.

#### Postoperative care

**Figure 2:**
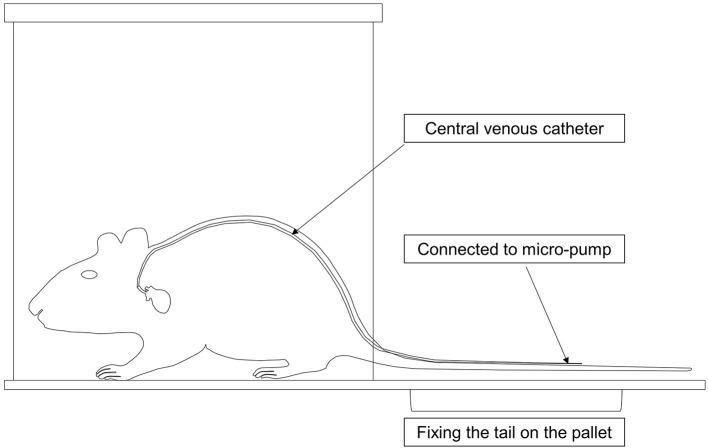
Mode chart of the total parenteral nutrition (TPN) device in mice. The catheter was threaded subcutaneously from the neck of the mouse along the back to the tail, where the tail was fixed onto the pallet. The mice were then placed into clean cages, and the catheter was connected to a multichannel micro-pump for TPN.

The mice were placed in clean mouse cages and kept warm on a 37 °C warming table. The jugular vein catheter was connected to a multichannel infusion pump delivering intravenous injection of TPN at a rate of 0.167 ml/h (Figure 2). A measure of 0.5 ml of normal saline was injected into the catheter every day to ensure its patency.

### EN model

After anesthesia, the abdominal cavity was entered layer by layer through a median incision in the upper abdomen, and the mouse stomach was identified using atraumatic forceps, and a ~0.3 cm stoma was made on the side of the greater curvature of the stomach near the pylorus. A medical silicone tube (Silastic 60-011-05) was inserted and gently advanced about 2 cm into the upper duodenum. After confirming the absence of slippage, a purse-string suture was made at the gastrostomy. Then the silicone tube was reinforced with an additional suture on the abdominal wall muscle.

### Parenteral and enteral nutrition

All mice were maintained for 7 days after surgery. Mice assigned to the TPN group receive a continuous infusion of TPN solution, starting at 0.25 ml/h (first day of infusion, 6 ml/day) and thereafter increasing to 0.3 ml/h (second and third days of infusion, 7.2 ml/day), then 0.32 ml/h (fourth through seventh days of infusion, 7.7 ml/day). Enteral nutrition suspension (TPF) was administered via gastrostomy catheter (16) in the EN group for 7 days.

### Dosage information

The dose in TPN was determined based on nutritional requirements, including protein (provided as an amino acid solution, 4 kcal/g) and non-protein calorie-providing macronutrients such as carbohydrates (supplied as glucose, 3.4 kcal/g) and lipids (supplied as a lipid emulsion, ≈10 kcal/g). The total calories provided by the TPN solution were calculated by summing the calories from non-protein and protein sources. Mice in the TPN group received an isocaloric solution (150 kcal/100 ml; Table 1). Enteral nutrition (NUTRICIA INTERNATIONAL B.V., Co., LTD) was administered with the same amount of nitrogen and calories (150 kcal/100 ml) as the TPN group in TPF.

**Table 1:** TPN formulation.

**Nutrient source**	**Amount per 100 ml**
Amino acids (Aminoven 15%, Fresenius Kabi)	32 ml (4.8 g protein)
Glucose (70%)	44.5 ml (31.2 g sugar)
Lipids (Intralipid 20%, Fresenius Kabi)	12 ml (2.4 g fat)
Trace elements (Addaven, Fresenius Kabi)	0.5 ml
Multivitamins (Addamel, Fresenius Kabi)	0.1 ml

### Euthanasia and dissection

All animals were euthanized using 20% CO_2_ at a flow rate of 5.8 L/min on the seventh day after surgery. Two segments of the terminal ileum, each approximately 2 cm in length, were selected from a distance of approximately 3 cm from the ileocecal region. One segment was fixed in 4% formaldehyde, dehydrated, embedded, and sectioned for subsequent tests, while the other segment was fixed in 10% formaldehyde.

### Sample analysis

Tissue samples were fixed in formalin, dehydrated, and embedded in paraffin. Hematoxylin and eosin (H&E) staining was performed to assess intestinal histology. Immunohistochemistry (IHC) and immunofluorescence (IF) were performed on intestinal tissues fixed in 10% formalin. The proliferating cell nuclear antigen (PCNA) abundance was assessed by immunofluorescence (IF), lysozyme, ZO-1, and occludin were assessed by immunohistochemistry (IHC). Evaluation and semi-quantitative analysis of IHC detection were conducted using ImageJ software. The average gray value (staining intensity) of positive cells and the percentage of positive area (staining area) were jointly used as the measurement indicators of IHC, and finally, four scores were given: high positive (3+), positive (2+), low positive (1+), and negative (0). Goblet cell counts were assessed using Periodic Acid-Schiff (PAS) staining.

### Statistical analysis

Statistical analysis was conducted using SPSS 25.0 statistical software (SPSS Inc., Chicago, IL, USA). The data all show a normal distribution. Statistical analyses were performed as follows: the experiment was repeated at least three times, and the experimental data were expressed as mean ± standard deviation (mean ± SD), with comparisons between two groups using Student's *t*-test. Data were presented as mean ± SD. *P*-values of < 0.05 were considered statistically significant.

## Results

### General description

The weight of the mice was recorded before the start of the study and after the mice were euthanized. In the TPN group, the weights at the beginning and end of the study were 23.9 ± 1.7 g and 24.1 ± 1.6 g, respectively. In the EN group, the corresponding values were 24.0 ± 1.9 g and 24.5 ± 1.6 g (*P* > 0.05). All animals survived for 7 days post-intervention until the experimental endpoint was reached.

### TPN leads to intestinal villus atrophy

**Figure 3:**
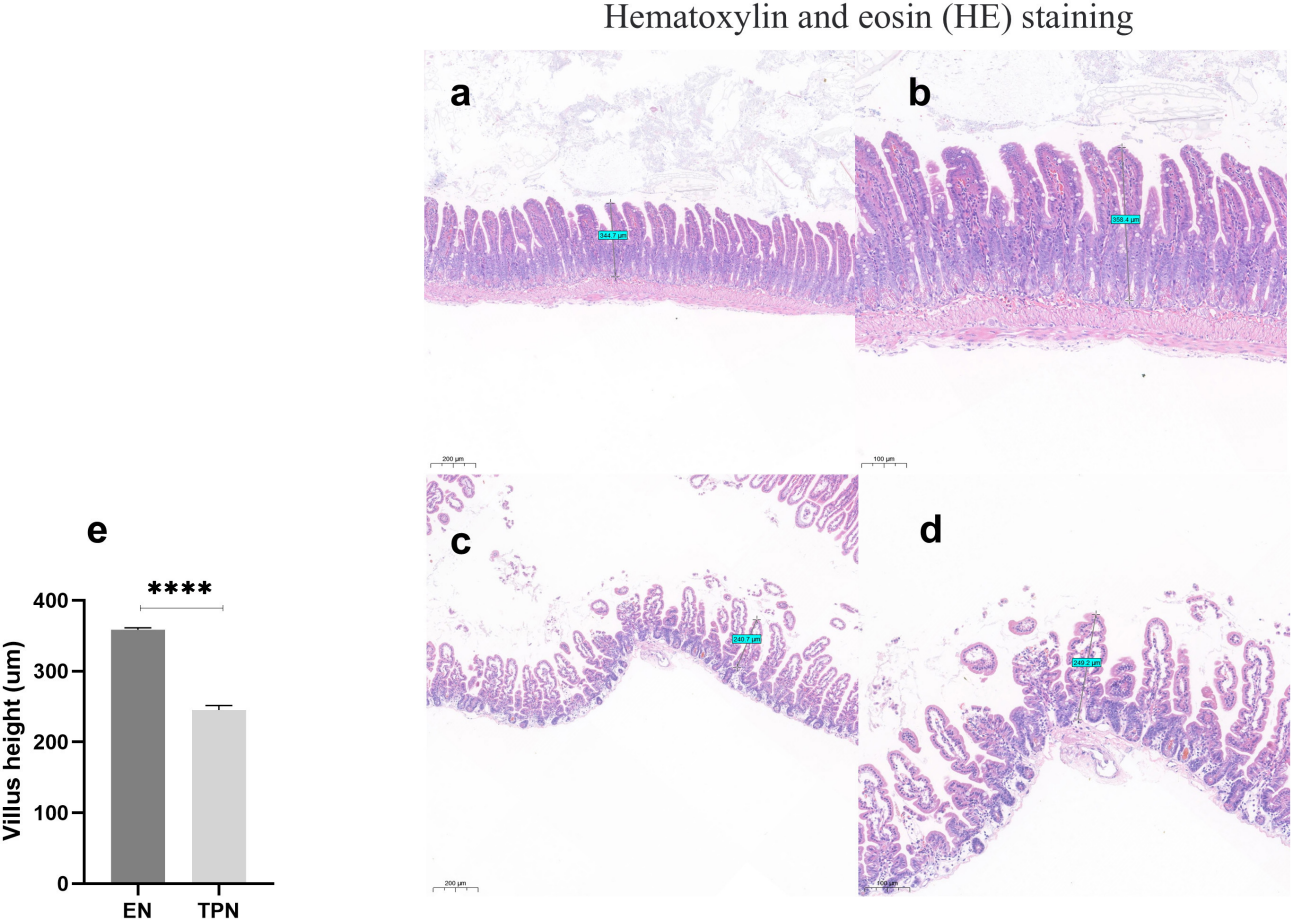
Representative image of harvested intestine from enteral nutrition (EN) and total parenteral nutrition (TPN) mice. The height of the intestinal villi was measured. TPN leads to intestinal villus atrophy. EN group **(a, b), (a)** Scale bar, 100 mm, **(b)** Scale bar, 50 mm; TPN group **(c, d), (c)** Scale bar, 100 mm, **(d)** Scale bar, 50 mm; **(e)** the height of intestinal villi. ****P < 0.0001.

The intestinal tissue was stained with HE, and the height of intestinal villi was measured. The results demonstrated that the height of intestinal villi in the TPN group was significantly reduced compared to the EN group (*P* < 0.0001; Figure 3).

### TPN reduces intestinal proliferation

**Figure 4:**
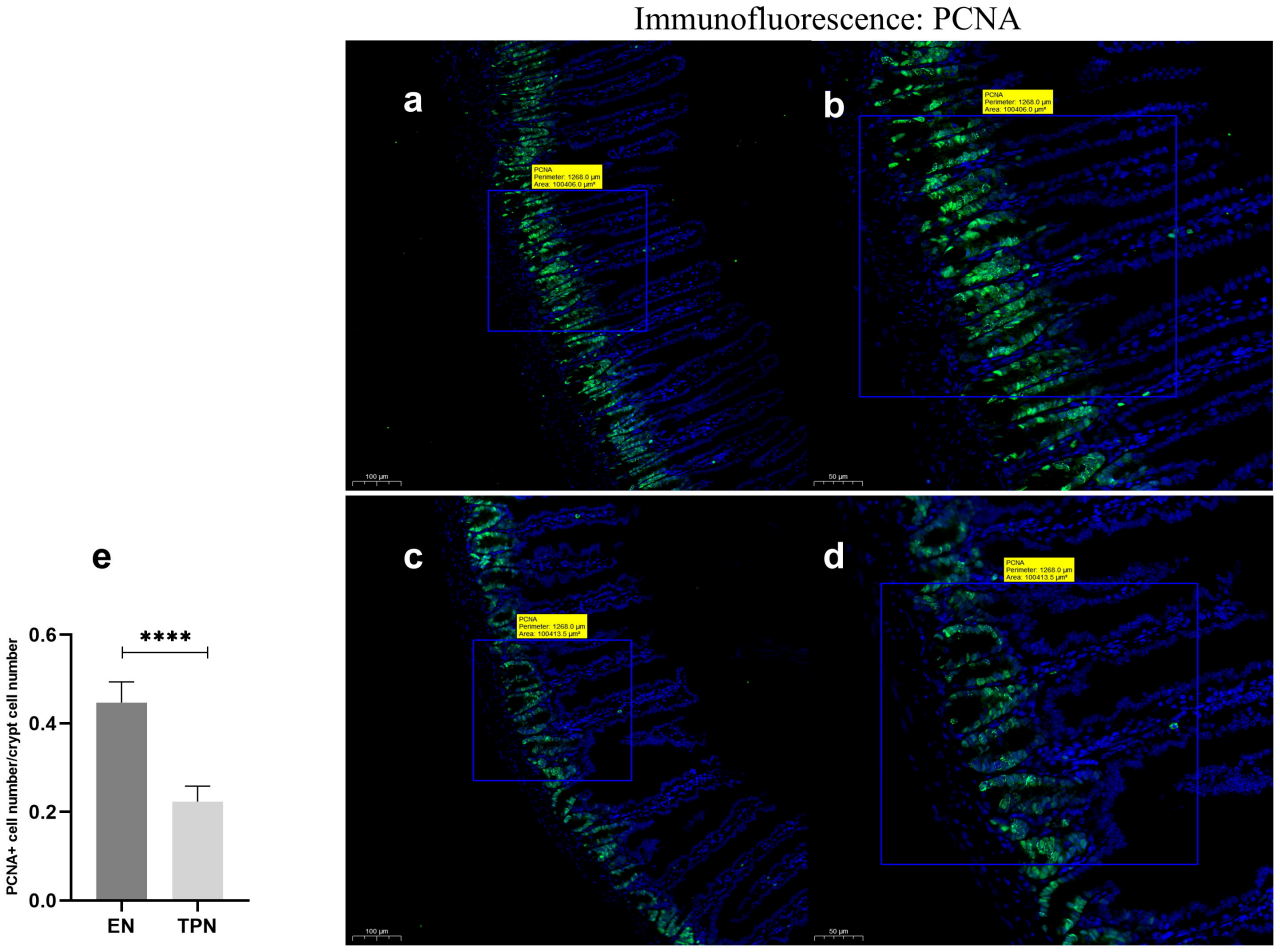
Proliferating cell nuclear antigen (PCNA) immunofluorescence staining was performed to measure IEC proliferation. TPN leads to diminished proliferation of intestinal epithelial cells (IEC). Proliferation index was calculated as the ratio of PCNA-positive cell number to total cell number in each crypt; EN group **(a, b)**, **(a)** Scale bar, 100 mm, **(b)** Scale bar, 50 mm; TPN group **(c, d), (c)** Scale bar, 100 mm, **(d)** Scale bar, 50 mm; **(e)** proliferation index for each group. ****P < 0.0001.

The proliferating cell nuclear antigen (PCNA) abundance was assessed by immunofluorescence (IF). It was found that the abundance of PCNA was significantly lower in the TPN group compared to the EN group (*P* < 0.0001; Figure 4).

### TPN causes a decrease in the number of intestinal goblet cells

**Figure 5:**
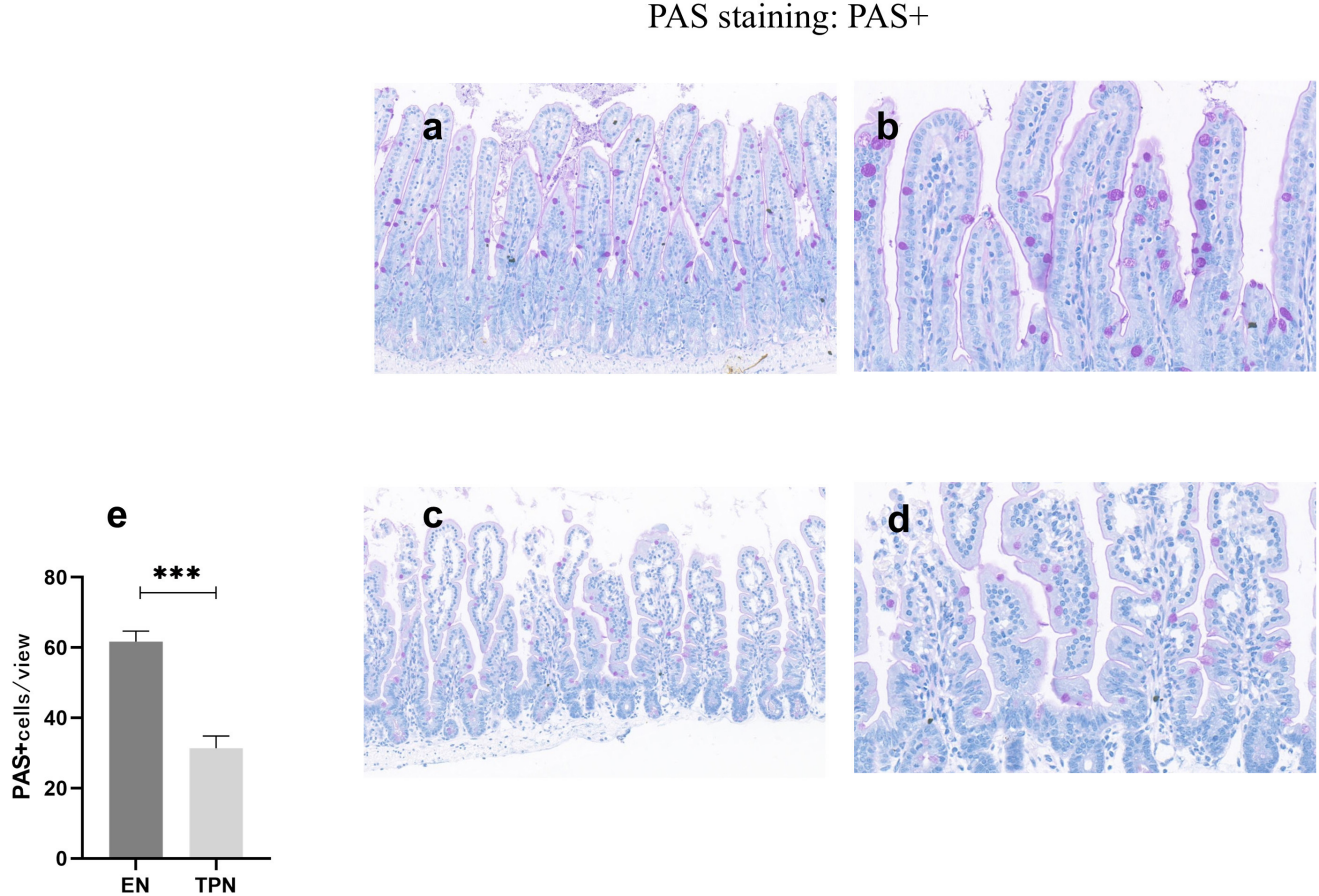
PAS was performed to measure the number of intestinal goblet cells. TPN causes a decrease in the number of intestinal goblet cells. EN group **(a, b), (a)** Scale bar, 100 mm, **(b)** Scale bar, 50 mm; TPN group **(c, d), (c)** Scale bar, 100 mm, **(d)** Scale bar, 50 mm; **(e)** the number of goblet cells for each group. ***P < 0.001.

After glycogen PAS staining, the cells were observed under light microscopy. The cells stained with red glycogen could be seen as goblet cells under high magnification. Three regions were chosen randomly to calculate the number of goblet cells and compared. The results showed that the number of goblet cells in the TPN group was significantly lower than in the EN group (*P* < 0.001; Figure 5).

### TPN causes a decreased function of intestinal Paneth cells

**Figure 6:**
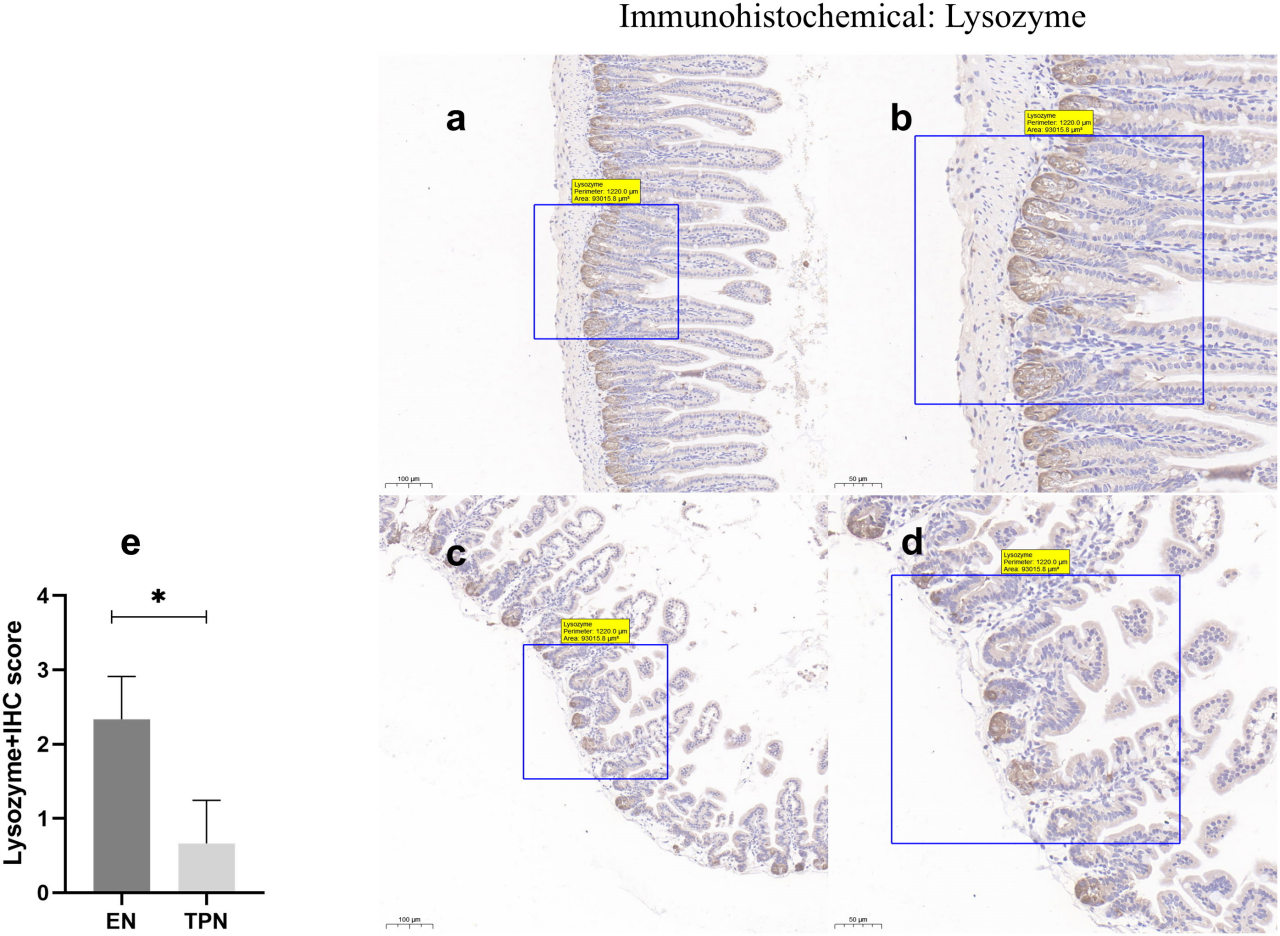
Lysozyme immunohistochemical was performed to measure the function of intestinal Paneth cells. EN group **(a, b), (a)** Scale bar, 100 mm, **(b)** Scale bar, 50 mm; TPN group **(c, d)**, **(c)** Scale bar, 100 mm, **(d)** Scale bar, 50 mm; **(e)** the IHC score of Lysozyme positive cells for each group. *P < 0.05.

Under light microscopy, there was no significant difference in the number of Paneth cells between the two groups. The Lysozyme+ cells in the EN group had more intensely stained granules, which were immune complexes of lysozyme proteins, while the mice in the TPN group had fewer granules and were significantly lighter in color than the control group (*P* < 0.0001; Figure 6).

### TPN disrupts tight junctions between intestinal epithelia

**Figure 7:**
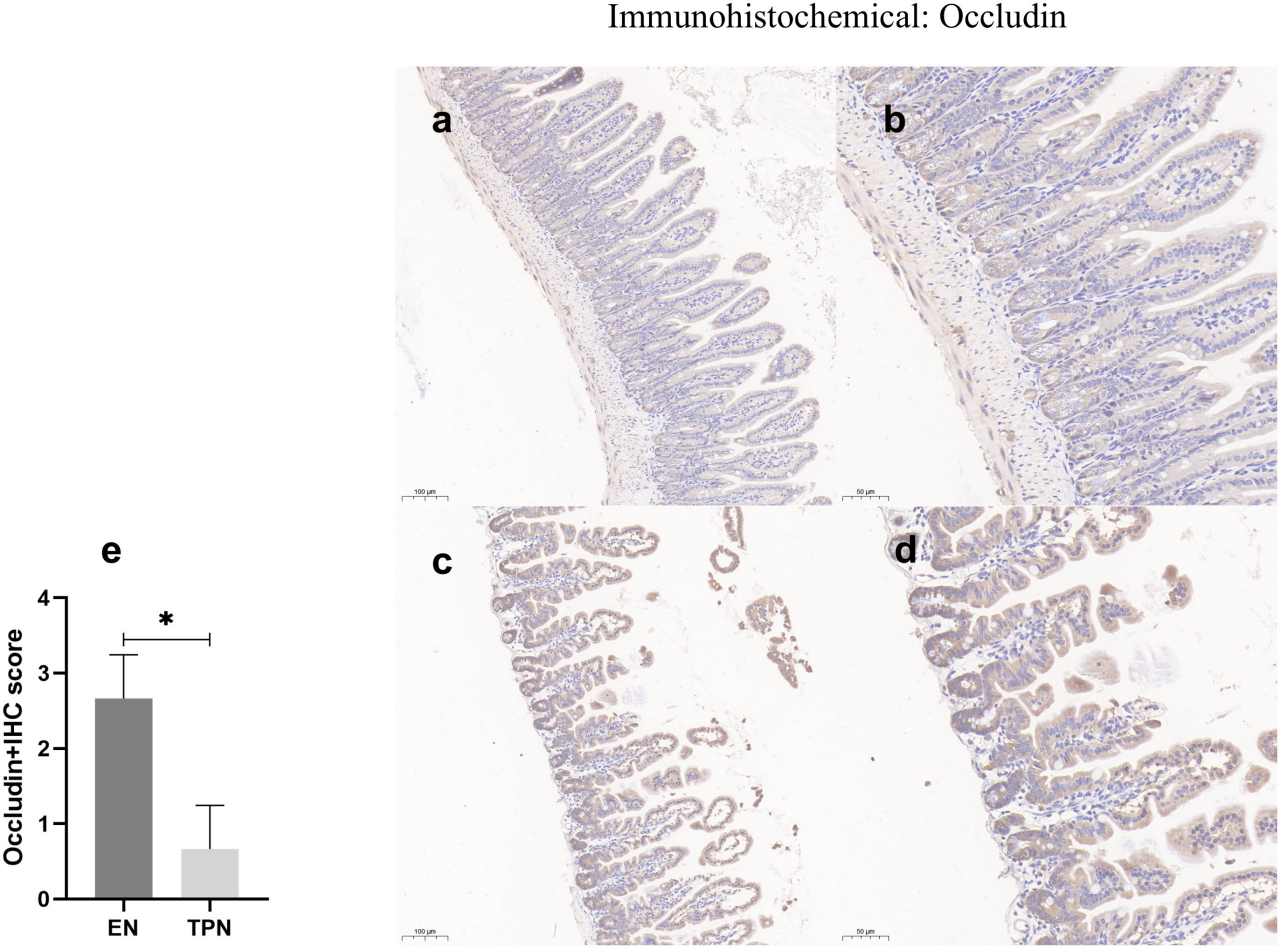
Occludin immunohistochemical was performed to measure the intestinal epithelial tight junction (TJ). Note the marked increase in the EN group and lower levels of intensity in the TPN group. EN group **(a, b), (a)** Scale bar, 100 mm, **(b)** Scale bar, 50 mm; TPN group **(c, d), (c)** Scale bar, 100 mm, **(d)** Scale bar, 50 mm; **(e)** the IHC score of occludin-positive cells for each group. *P < 0.05.

**Figure 8:**
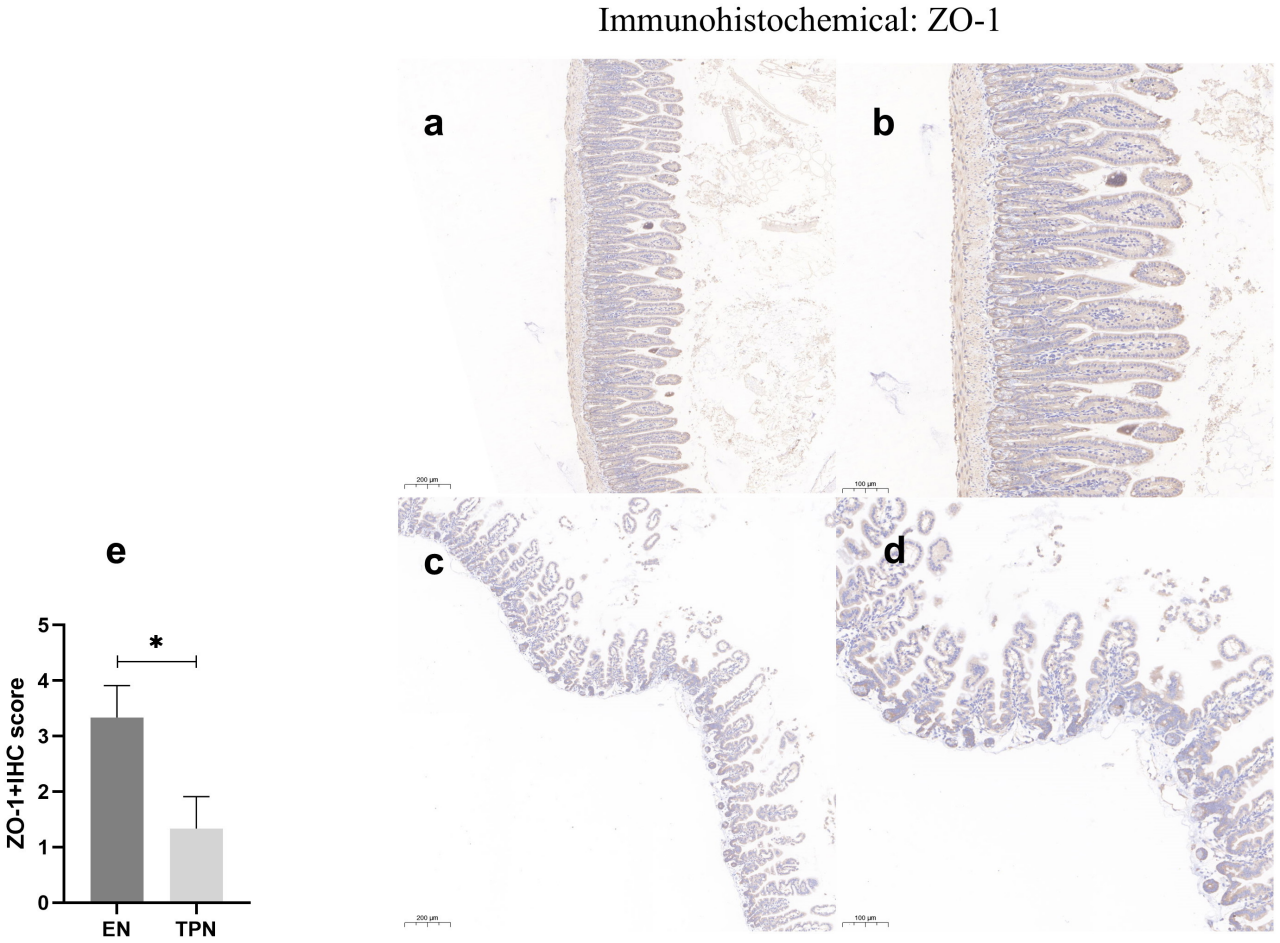
ZO-1 immunohistochemical staining was performed to measure the intestinal epithelial tight junction (TJ). Note the marked increase in the EN group and lower levels of intensity in the TPN group. EN group **(a, b), (a)** Scale bar, 100 mm, **(b)** Scale bar, 50 mm; TPN group **(c, d), (c)** Scale bar, 100 mm, **(d)** Scale bar, 50 mm; **(e)** the IHC score of ZO-1 positive cells for each group. *P < 0.05.

The epithelial tight junction (TJ) complex serves as a key structure of intestinal EBF, which keeps the intestinal epithelial cells tightly bound to each other and forms a selectively permeable membrane (10, 17). Several important proteins comprise the TJ complex, including Zonula occludens (ZO), occludin, and claudins (18, 19). TJ proteins block the paracellular passage of intraluminal contents such as pathogens, toxins, and nutrients. In this study, representative TJ proteins (ZO-1 and occludin) were evaluated by immunohistochemistry. In the intestinal epithelium of the EN group, ZO-1 and occludin along the apical were clearly expressed. In contrast, in the TPN group, the intercellular junctions among epithelial cells were almost lost (Figures 7, 8).

## Discussion and future directions

The intestinal epithelium is characterized by rapid renewal, with IECs undergoing complete renewal every 4–5 days (20). The intestinal epithelial layer consists of several different types of epithelial cells, including goblet cells and Paneth cells. Paneth cells, located at the base of the crypt, secrete αβ-1,4-N-acetylcarbamoyl hydrolase, a C-type lysozyme, which disrupts bacterial cell walls and, thus, impedes the entry of microorganisms into the intestinal lumen (21). Goblet cells secrete trefoil peptides, resistin-like molecule-β, and mucus, all of which play essential roles in epithelial layer homeostasis for intestinal epithelial defense and repair (17, 22). In this study, we found that epithelial proliferation and the number or function of intestinal epithelial cells—specifically Paneth cells and goblet cells, which help defend against intestinal microbiota—were significantly reduced in the TPN group compared to the EN group. This suggests that TPN compromises mucosal barrier function by impairing normal epithelial cell function. The relevant data are shown in Table 2 as follows.

**Table 2:** Expression of PCNA, lysozyme+, ZO-1, occludin, and PAS+ between the EN group and TPN group.

**Item**	**EN group (*n* = 10)**	**TPN group (*n* = 10)**	***P*-value**
PCNA (mean ± SD, ratio)	0.44 ± 0.09	0.21 ± 0.15	< 0.0001
Lysozyme+ (mean ± SD, score)	2.33 ± 0.57	0.66 ± 0.57	< 0.05
ZO-1 (mean ± SD, score)	3.33 ± 0.57	1.33 ± 0.57	< 0.05
Occludin (mean ± SD, score)	2.66 ± 0.57	0.66 ± 0.57	< 0.05
PAS+ (mean ± SD, Number of positive stained cells/view)	61.56 ± 1.76	31.03 ± 2.02	< 0.001

TPN provides life-sustaining nutrients to patients unable to tolerate EN-such as those with critically ill, perioperative bowel dysfunction, or short-bowel syndrome (10). However, a loss of EBF was observed in both *in vitro* and *in vivo* experiments of TPN (7, 12). This disruption of the epithelial barrier may contribute to the translocation of pathogenic intestinal bacteria and toxins into the circulation, resulting in infectious complications such as surgical site infections, sepsis, and pneumonia (23–25). Deficiencies in intestinal nutrition contribute to the loss of EBF, and delivery by the intestinal route has been shown to mitigate the loss of EBF (26). The loss of EBF has been associated with a variety of factors, including increased IEC apoptosis, reduced IEC proliferation, and decreased expression of TJ-associated proteins (9, 10, 27). Furthermore, it is coupled with changes in the ultrastructure of the epithelial TJ, characterized by a decrease in the number of TJ strands and a decrease in the depth of the main TJ mesh, together with the occurrence of the TJ strand as discontinuous (28). Consistent with previous findings, the present study also discovered that the expression of occludin and ZO-1, important tight junction proteins, was significantly lower in the TPN group than in the EN group, indicating that TPN may affect the synthesis of tight junction proteins between epithelium.

Previous studies have shown that fever and inflammation after trauma or surgery are frequent and greatly impact patient clinical outcomes. The origin of the infection is likely to be related to intestinal flora displacement due to postoperative intestinal barrier damage. After the completion of the modeling process, we demonstrated that TPN may cause a reduction of small intestinal epithelial cell proliferation and impaired intestinal barrier function when compared with EN. Therefore, this study provides a complete, reliable, and optimized mouse model of the effects of TPN and EN on intestinal mucosal barrier function.

## Conclusion

In this study, we focused on the mold-making process and detailing methodology, using mice to study the effects of TPN and EN on intestinal barrier function. The results presented a high success rate in model establishment and consistent experimental outcomes, largely due to the practical advantages of mice, including their availability and ease of handling. Therefore, using mice as animal models for TPN and EN research is worth promoting in future studies exploring the impact of TPN on intestinal barrier function.

## Data Availability

The datasets presented in this study can be found in online repositories. The names of the repository/repositories and accession number(s) can be found in the article/supplementary material.
